# Supplementary material for: Behavior of Mebendazole during NF/RO Adsorption and Photolysis
Source: Membranes (Basel). 2022 Sep 15;12(9):888. doi: 10.3390/membranes12090888 (PMC9503556; doi:10.3390/membranes12090888)
Supplement: Supplementary file 1 [file membranes-12-00888-s001.zip › membranes-1908151-supplementary.pdf]

Supplementary information

Behavior of Mebendazole during NF/RO Adsorption and Photolysis

Bruna Babić [1], Darko Andrić [1], Anamarija Farkaš [2], Dragana Vuk [3], Danijela Ašperger [1] and Davor Dolar [4,*]

[1] Department of Analytical Chemistry, Faculty of Chemical Engineering and Technology, University of Zagreb, Marulićev trg 19, 10000 Zagreb, Croatia
[2] The Institute for Development and International Relations, Ljudevita Farkaša Vukotinovića 2, 10000 Zagreb, Croatia
[3] Department of Organic Chemistry, Faculty of Chemical Engineering and Technology, University of Zagreb, Marulićev trg 19, 10000 Zagreb, Croatia
[4] Department of Physical Chemistry, Faculty of Chemical Engineering and Technology, University of Zagreb, Marulićev trg 19, 10000 Zagreb, Croatia
* Correspondence: dolar@fkit.hr; Tel.: +385-1-4597-233

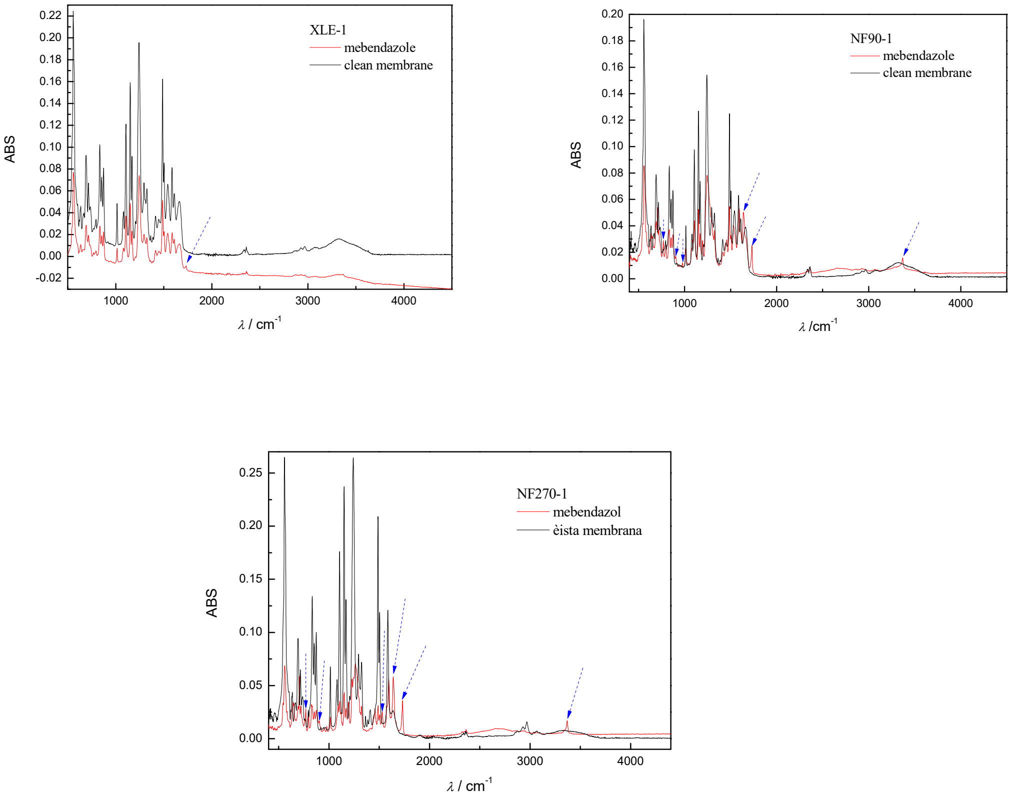

Figure S1. FTIR spectra of RO/NF membranes before and after the treatment of mebendazole solution

a)

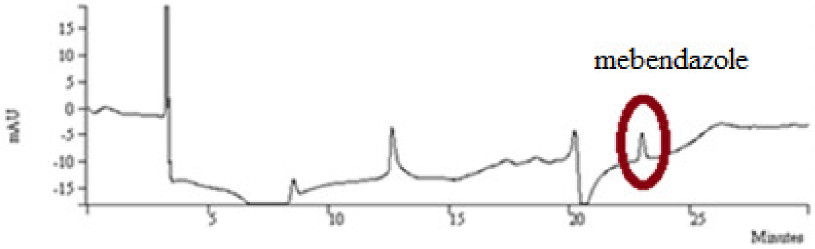

b)

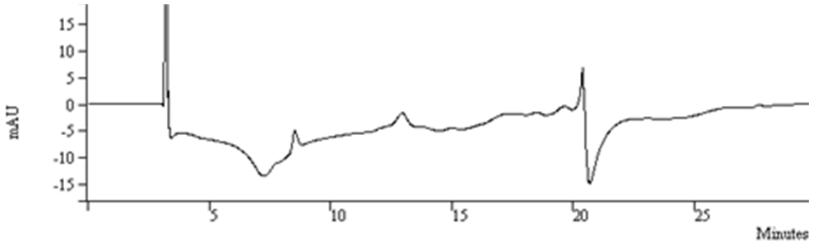

Figure S2. Chromatogram of 1 mg/L mebendazole solution: a) initial solution before column adsorption and b) after column adsorption at *h*(GAC) = 40 cm

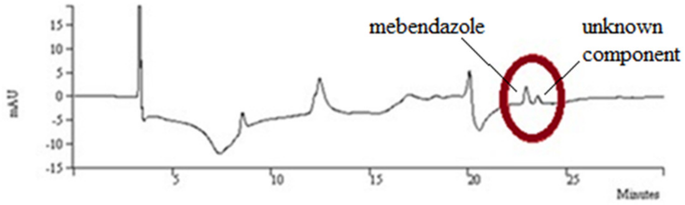

Figure S3. Chromatogram of 3 mg/L mebendazole solution and unknown component after photolysis

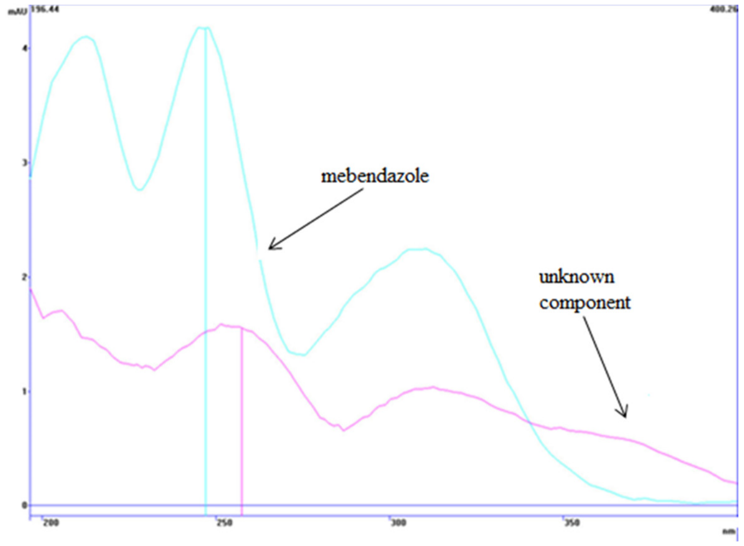

Figure S4. Absorption spectrum of mebendazole and unknown component

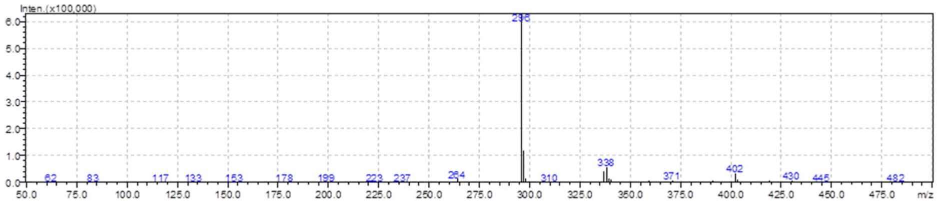

Figure S5. Mass spectrum of mebendazole

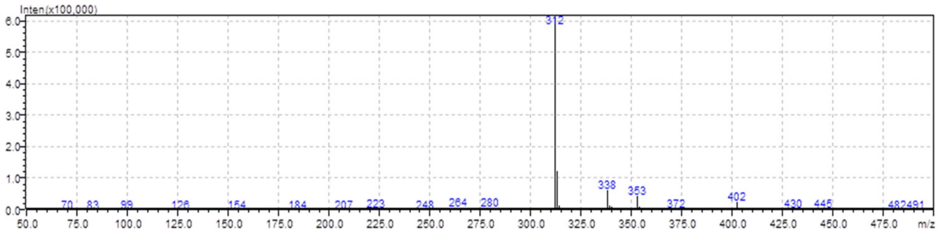

Figure S6. Mass spectrum of the degradation or transformation product
